# Migration of all-polyethylene compared with metal-backed tibial components in cemented total knee arthroplasty: A randomized controlled trial

**DOI:** 10.1080/17453674.2018.1464317

**Published:** 2018-05-01

**Authors:** Koen T Van Hamersveld, Perla J Marang-Van De Mheen, Rob G H H Nelissen, Sören Toksvig-Larsen

**Affiliations:** a Department of Orthopaedics, Leiden University Medical Center, Leiden;; b Medical Decision Making, Leiden University Medical Center, Leiden;; c Department of Orthopaedics, Hässleholm Hospital, Hässleholm, Sweden and Department of Clinical Sciences, Lund University, Lund, Sweden

## Abstract

**Background and purpose — With a rapidly increasing population in need of total knee arthroplasty (TKA), there is renewed interest in cost-saving all-polyethylene designs. Differences between metal-backed and all-polyethylene designs in initial component migration assessed by radiostereometric analysis (RSA), a proven predictor for late aseptic loosening, have been scantily reported. The purpose of this study was to compare implant migration and clinical outcomes of all-polyethylene tibial components versus metal-backed trays of similar geometrical shape.**

**Patients and methods — In this randomized controlled trial, 59 patients received a cemented Triathlon condylar-stabilizing implant (Stryker, Mahwah, NJ, USA) with either an all-polyethylene (n = 29) or a metal-backed tibial component (n = 30). RSA measurements and clinical scores (the Knee Society Score, Forgotten Joint Score, and Knee Osteoarthritis and Injury Outcome Score) were evaluated at baseline and postoperatively at 3, 12, and 24 months. A linear mixed-effects model was used to analyze the repeated measurements.**

**Results — A statistically significant difference in mean migration after 2 years was found in favor of the all-polyethylene group, with a mean maximum total point motion of 0.61 mm (95% CI 0.49–0.74) versus 0.81 mm (95% CI 0.68–0.96) for the cemented group (p = 0.03). However, this difference was smaller and not statistically significant after post hoc adjustment for surgeon effect. Both groups showed comparable improvements on all clinical outcome scores over time.**

**Interpretation — The Triathlon all-polyethylene tibial component showed less migration, suggesting a lower risk of late loosening as compared with its metal-backed counterpart. However, the found surgeon effect warrants further investigation.**

Metal-backed tibial components in total knee arthroplasty (TKA) have primarily been used since their introduction in the late 1970s, as clinical results were superior to the first generation of all-polyethylene tibial components (Gioe and Maheshwari 2010). With a rapidly increasing population in need of knee arthroplasty, the associated healthcare costs are expected to rise exponentially (Kurtz et al. 2007). This triggered renewed interest in all-polyethylene designs as manufacturing such implants costs 20% to 50% less (Gioe and Maheshwari 2010). Meta-analyses comparing modern all-polyethylene and metal-backed tibial components show equivalent results in terms of risk for revision and clinical scores, yet all-polyethylene designs are still rarely used (Voigt and Mosier 2011, Nouta et al. 2012, Voss et al. 2016).

Given that first-generation all-polyethylene designs often failed secondary to aseptic loosening, many surgeons today are reluctant to use all-polyethylene components (Voss et al. 2016). More evidence is thus needed on the fixation of today’s all-polyethylene designs, preferably by radiostereometric analysis (RSA). None of the few RSA studies published to date has shown superiority of metal-backed designs over all-polyethylene designs (Adalberth et al. 1999, 2000, 2001, Norgren et al. 2004, Hyldahl et al. 2005a, b, Muller et al. 2006). Moreover, Hyldahl et al. (2005a) found lower initial migration in AGC all-polyethylene components (Biomet, Warsaw, IN, USA). They hypothesized that these—to some degree elastic—components may partly absorb eccentric forces, while the more rigid metal-backed design is thought to transform asymmetric load throughout the entire component, inducing adverse tensile forces.

With further improvements in implant design and quality of materials over the past decades, the clinical performance of either design could nowadays well outperform the other. We therefore conducted a randomized controlled trial in which we compared implant migration and clinical performance of a relatively new all-polyethylene tibial component with a similarly designed metal-backed tray of the Triathlon total knee prosthesis (Stryker, Mahwah, NJ, USA). The femoral component of this prosthesis is designed to rotate about a single axis during flexion, which should provide ligament isometry and a larger contact area throughout the range of motion (Wolterbeek et al. 2012). Any remaining peripheral peak stresses that could compromise implant fixation might be better absorbed by the more elastic all-polyethylene design. Based on this theory, we hypothesized the all-polyethylene design to show less implant migration as compared with its metal-backed counterpart. 

## Patients and methods

This randomized controlled trial was conducted in Hässleholm Hospital, Sweden. All consecutive patients with primary osteoarthritis scheduled to undergo TKA between June 2014 and November 2014 were asked to participate. The main exclusion criterion was when regular postoperative visits for RSA and clinical evaluations were considered impractical, due to, for example, long travel time. A computer-generated randomization list was created by the study monitor (1:1 ratio with a block size of 20). Opening the sequentially numbered, opaque, sealed envelopes only on the day of surgery ensured concealment of treatment allocation. Patients remained blinded throughout follow-up, which was not the case for surgeons and observers performing clinical follow-up due to the marked difference in radiographic appearance between implant designs.

### Prosthesis and surgical technique

Surgeries were performed by 2 experienced surgeons using standardized techniques according to the Triathlon knee system surgical protocol. All patients received condylar-stabilizing (i.e., with a deep-dished polyethylene insert) cruciate-retaining Triathlon total knee prostheses indicated for cemented fixation, with either modular metal-backed tibial components using highly cross-linked polyethylene inserts or monoblock all-polyethylene tibial components of similar geometrical shape made from conventional N2/Vac ultra-high molecular weight polyethylene. Both surgeons used a standard midline incision and medial parapatellar arthrotomy, preserved the posterior cruciate ligament and used pulsatile lavage prior to applying SmartSet GHV bone cement (DePuy CMW, Blackpool, UK) with the tibial keel uncemented in all procedures. No tourniquet was used and patellae were not resurfaced. For RSA purposes, 8 tantalum markers were inserted into the proximal tibial metaphysis and 5 markers were inserted (proximally) in the polyethylene insert at standardized positions (0.8 mm diameter; RSA Biomedical, Umeå, Sweden). Postoperatively, low molecular heparin (enoxaparin intramuscular 40 mg/day) was prescribed for 10 days and patients were stimulated to mobilize with immediate full weight-bearing.

### Follow-up

Preoperatively, the Knee Society Score (KSS), Knee injury and Osteoarthritis Outcome Score (KOOS), and hip–knee–ankle angle (HKA) measurements (with varus <180°) were assessed. Postoperative evaluations including RSA radiographs were performed on the first day after surgery. Subsequent RSA and clinical examinations including the KSS, KOOS, and the Forgotten Joint Score (FJS) were scheduled at 3 months, 1 year, and 2 years after surgery. The FJS questionnaire is a relatively new outcome measurement with increased discriminatory power in especially well-performing patients (i.e., able to detect small differences between good, very good, and excellent patients) (Behrend et al. 2012, Thomsen et al. 2016). HKA measurements were repeated at 3 months’ follow-up.

### Radiostereometric analysis

To ensure similar measurement techniques between the radiolucent all-polyethylene design and the metal-backed design, marker-based RSA analysis was performed using the tantalum markers inserted at standardized positions in both designs. RSA radiographs were made in supine position with the knee in a calibration cage (Cage 10, RSA Biomedical, Umeå, Sweden) and analyzed using MB-RSA software version 4 (RSAcore, LUMC, Leiden, the Netherlands). The precision of the RSA setup was determined by taking “double examinations” at the 1-year follow-up and, as no actual migration is expected within the few minutes of time between examinations, is expressed as the upper limits of the 95% confidence interval (CI) around zero motion (ISO 16087:2013(E) 2013). Levene’s test for equality of variances was applied to test for differences in precision between modular (metal-backed) and monoblock (all-polyethylene) components. Positive directions along and about the orthogonal axes are according to the right-hand screw rule (Valstar et al. 2005). Migration was described as translation of the geometric center of the prosthesis markers and rotation about the geometric center of gravity. The maximum total point motion (MTPM), which is the length of the translation vector of the marker or virtual marker in a rigid body that has the greatest migration, was used as the primary outcome measure (ISO 16087:2013(E) 2013). The direct postoperative RSA examination served as the reference for the migration measurements. Besides migration on a group level, the number of individual components showing “continuous migration,” defined by Ryd et al. (1995) as an increase in MTPM of 0.2 mm or more in the second postoperative year indicating an increased risk for aseptic loosening, are also reported. Marker stability and scatter values were within the limits of RSA guidelines (Valstar et al. 2005).

### Sample size

Earlier RSA studies using the Triathlon total knee prosthesis have shown measurement errors of less than 0.25 mm (Molt et al. 2016). With an alpha of 0.05 and power of 80%, 17 patients were needed to detect a mean difference larger than 0.25 mm. To account for loss to follow-up, 30 patients were randomized to each group.

### Statistics

All outcome measurements were analyzed according to the intention-to-treat principle using a linear mixed-effects model. This method accounts for the correlation of the repeated measurements in patients and deals effectively with missing values (Ranstam et al. 2012). Treatment, time, and the interaction of time with treatment were modeled as fixed factors, patients were included as a random factor and a compound symmetry covariance structure was assumed. MTPM was log-transformed during statistical modeling to obtain a normal distribution, computed as log10(MTPM +1). Additionally, we conducted a post hoc sensitivity analysis to determine the effect of possible confounders on treatment by adding any baseline characteristic that was by chance not evenly distributed between groups as variables to the model, as well as their interaction with time. To analyze differences in mean migration along and about each orthogonal axis, only absolute values were used (as calculating the resultant of positive and negative displacement vectors requires all vectors to act on the same prosthesis) (Derbyshire et al. 2009). These outcome parameters were also log-transformed in a similar manner to MTPM to obtain normal distribution. Significance was set at p < 0.05 (IBM SPSS Statistics 23.0; IBM Corp, Armonk, NY, USA).

### Ethics, registration, funding, and potential conflicts of interest

The trial was performed in compliance with the Declaration of Helsinki and Good Clinical Practice guidelines. The study was approved by the Regional Ethical Review Board in Lund prior to enrollment (entry no. 2013/434) and registered at isrctn.com (ID: ISRCTN04081530). Informed consent was obtained from all patients. Reporting of the trial was in accordance with the CONSORT statement. Stryker provided funds in support of the costs associated with RSA radiographs and extra clinical follow-up examinations. The sponsor did not take any part in the design, conduct, analysis, and interpretations stated in the final manuscript. 

## Results

**Figure 1.**
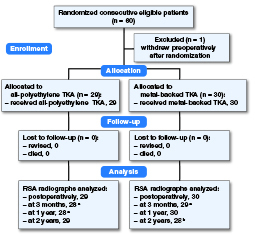
CONSORT flow diagram. TKA = total knee arthroplasty. **^a^**Missed follow-up; **^b^**Technical reasons, clinical follow-up only.

60 patients were randomized of whom 1 patient withdrew from the study prior to surgery. This patient was not replaced, resulting in 29 patients receiving the allocated all-polyethylene components and 30 patients receiving allocated metal-backed components (Figure 1). At 2-year follow-up, the RSA images of 2 patients with metal-backed components could not be analyzed for technical reasons (1 stereo image had too few reference cage markers and 1 stereo image did not match). Both patients had low migration up to 1 year (MTPM <0.3 mm) and at 2-year follow-up no signs of loosening on conventional radiographs and good clinical scores. Due to chance, more females were randomized to the all-polyethylene group and surgeries were not evenly distributed between the two surgeons (Table 1). Other than that, groups were comparable at baseline.

**Table 1.** Baseline demographic characteristics

Outcome	All-polyethylene (n = 29)	Metal-backed (n = 30)
Age, mean (SD)	69 (5.5)	68 (5.6)
BMI, mean (SD)	28 (4.2)	29 (3.0)
Female sex, n	22	13
Ahlbäck’s classification, n		
II	6	10
III	21	19
IV	2	1
HKA preoperative, n		
Varus (< 177°)	22	25
Neutral (177–183°)	5	3
Valgus (> 183°)	2	2
HKA postoperative, n		
Varus (< 177°)	4	7
Neutral (177–183°)	19	22
Valgus (> 183°)	6	1
Surgeon 1, n performed	20	14
Surgeon 2, n performed	9	16

HKA: hip–knee–ankle angle.

## Radiostereometric analysis

The precision of the RSA setup was determined by making double examinations in 48 patients (of which 22 patients had metal-backed components) at one-year follow-up. The precision (expressed as the CI around zero motion) of transverse, longitudinal, and sagittal axis translation was 0.09 mm, 0.13 mm, and 0.11 mm, respectively; and of transverse, longitudinal, and sagittal rotation 0.15°, 0.12°, and 0.11°, respectively. There were no differences in precision between groups (p > 0.15 for all translations and rotations).

**Figure 2.**
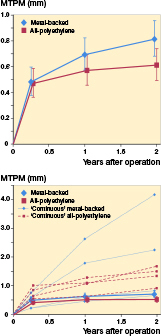
RSA analysis results of maximum total point motion (MTPM). Top: mean and 95% confidence interval for the groups; bottom: mean and 95% confidence interval for the same groups excluding 8 individual components showing continuous migration of >0.2 mm in the second postoperative year. These individual components are illustrated as 4 dashed blue lines (metal-backed) and 4 dashed red lines (all-polyethylene).

The results of the primary outcome MTPM showed a higher mean MTPM of 0.81 mm (CI 0.68–0.96) for the metal-backed group versus 0.61 mm (CI 0.49–0.74) for the all-polyethylene group after 2 years’ follow-up (p = 0.03, Table 2). In both groups, 4 prostheses showed continuous migration in the second postoperative year, ranging from 0.2 mm up to 1.5 mm (Figure 2). Most components showing continuous migration still had MTPM values <1.5 mm at 2-year follow-up (Figure 2). The other RSA parameters revealed similar translations and rotations between groups at 2-year follow-up except for sagittal translation; the mean translation in the all-polyethylene group was 0.25 mm (CI 0.17–0.34) versus 0.43 mm (CI 0.34–0.52) for the metal-backed group (p = 0.006) (Table 3, see Supplementary data).

**Table 2.** RSA migration analysis of mean maximum total point motion (logMTPM values are back-transformed in original scale in millimeters), as provided by the mixed-effects model

	All-polyethylene	Metal-backed	
Time	mean (95% CI)	mean (95% CI)	p-value
3 months	0.47 (0.36–0.59)	0.48 (0.38–0.60)	
1 year	0.57 (0.46–0.69)	0.69 (0.57–0.82)	
2 years	0.61 (0.49–0.74)	0.81 (0.68–0.96)	0.03

In the post hoc sensitivity analysis (adjusting for a possible effect of the unevenly distributed covariates sex and surgeon), a statistically significant surgeon effect was found on migration; the mean logMTPM difference between surgeons at 2-year follow-up was 0.13 (CI 0.09–0.17, p < 0.001); sex had no statistically significant effect on migration (Table 4, see Supplementary data). Although all-polyethylene components showed on average less migration in both surgeon groups, the difference with metal-backed components was, in contrast with the primary analysis, not statistically significant anymore when adjusting for the surgeon effect (p = 0.2) (Figure 3 and Table 4, see Supplementary data).

## Clinical results and adverse events

The KSS score and all patient-reported outcome scores (KOOS and FJS) showed comparable improvements over time between groups (Table 5, see Supplementary data).

Several adverse events occurred (all in patients of the metal-backed group, except for the last patient described below). 1 patient suffered from peroneal nerve dysfunction directly postoperatively, which partially resolved. 2 venous thromboembolisms occurred within 3 months (1 deep-vein thrombosis and 1 pulmonary embolism) requiring temporary pharmacologic treatment. 1 patient experienced persistent anterior knee pain with patellar maltracking for which a medial patellofemoral ligament reconstruction was performed 14 months after the primary surgery (all components remained in situ). The patient continued to participate in the study showing moderate clinical scores at 2-year follow-up. Lastly, 1 patient (a 67-year-old female with an all-polyethylene component) sustained a supracondylar femur fracture of the ipsilateral leg following a fall accident 15 months after the primary surgery. She was initially treated using a lateral distal femoral locking plate, but this was converted to an intramedullary nail due to plate failure after 2 months. At 2 years’ follow-up, the patient and her knee functioned well with excellent clinical scores, no signs of loosening of the femoral component and a stable tibial component migration pattern similar to the group average. 

## Discussion

The results of the primary outcome of this study confirm our hypothesis that all-polyethylene components show statistically significantly lower migration after 2 years of follow-up compared with metal-backed trays of similar geometrical shape. However, smaller, non-significant differences were found after adjustment for surgeon effect in the post hoc analysis. As high initial migration is predictive for late aseptic loosening (Ryd et al. 1995, Pijls et al. 2012), our results suggests that by using a Triathlon all-polyethylene tibial component the risk of late loosening is at least comparable with, if not less than, that of its metal-backed counterpart.

Whereas the first-generation all-polyethylene TKA designs often failed due to loosening, our findings support a growing body of evidence that modern all-polyethylene designs are performing at least equally as well as metal-backed TKA designs (Voigt and Mosier 2011, Nouta et al. 2012, Voss et al. 2016). Previous RSA studies have shown all-polyethylene designs of various manufacturers to have comparable implant migration to its metal-backed counterpart (Adalberth et al. 1999, 2000, 2001, Norgren et al. 2004, Hyldahl et al. 2005a, b, Muller et al. 2006). Depending on the cementing technique, Hyldahl et al. (2005a, b) found comparable or lower migration of all-polyethylene components owing to the “teeter-totter” effect (i.e., tensile forces on the opposite side of the implant upon peripheral compressive loading). This adverse effect on migration was found to be greater when the tibial stem of the more rigid metal-backed tray was not cemented. As the tibial components in our study were only horizontally cemented, this could explain the higher migration of the metal-backed components in our study too.

Although there is a strong association between high initial migration and late loosening, it remains unclear how to optimally define “high” migration when comparing the performance of different implants (Henricson and Nilsson 2016). The found difference in mean MTPM suggests superiority of the all-polyethylene components over the metal-backed components. On the other hand, 4 components showed continuous migration in the second postoperative year in both groups, thus the number of individual components considered at risk for loosening is equal between groups. Furthermore, in the sensitivity analysis (adjusting for surgeon effect), results within each surgeon group appeared to be still in favor of the all-polyethylene components, but the differences were smaller and not significant anymore. The found surgeon effect highlights that, even today with all of the instrumentation available to promote standardization of surgical procedures, meticulous performance of each surgical step can improve the outcome, at least on a subclinical level. The results of the sensitivity analysis should, however, be regarded with caution due to multiple testing and an insufficient sample size for stratification by surgeon. It would be of interest if future RSA studies further explore this surgeon effect by randomizing patients to 2 or more surgeons using identical implants.

Most RSA studies have used maximum total point motion as the primary outcome to predict the occurrence of aseptic loosening (Grewal et al. 1992, Ryd et al. 1995, Pijls et al. 2012). Recently, however, Gudnason et al. (2017) advocated the use of other RSA parameters as the main predictor for loosening as MTPM has its limitations. One of the limitations is that one cannot infer the direction of migration of the MTPM values alone, resulting in uncertainty concerning the failure mechanism. But as motion implies a biological effect, which is expected to be greatest at the point of maximum motion (Valstar et al. 2005), merely expressing migration in fixed directions (e.g., anterior/posterior tilt) would in our opinion underestimate this effect in combined directions (e.g., subsidence into the medial-posterior tibial plateau with internal rotation). Another limitation of MTPM is that any movement between the polyethylene insert and the metal tray influences MTPM in marker-based RSA if polyethylene markers are used to represent the tibial component. Although improved locking mechanisms of modern fixed-bearing designs should prevent the insert from moving with respect to the metal tray, one should be aware of this phenomenon as previous studies have shown such movements to occur in older fixed-bearing designs, resulting in unreliable RSA measurements in the transverse plane (Rao et al. 2002, Nilsson et al. 2003, Hansson et al. 2005). It is therefore possible that the found difference is partly caused by movements between the modular components of the metal-backed design, rather than actual migration of the metal tray. One way to overcome this potential problem is to use model-based RSA measurements, but since all-polyethylene components are radiolucent, model-based RSA was only a possibility in the metal-backed trial arm. Given the known differences in precision between marker-based and model-based analysis (Kaptein et al. 2007), the current study was set up to use only marker-based RSA in both arms, rather than using different RSA methods in each arm. Furthermore, double examinations showed comparable precision between designs in all directions, indicating that the modular insert is most likely securely fixed within the tray. The influence of such movements on MTPM is therefore expected to be negligibly small.

In summary, a statistically significantly lower mean migration after 2 years was found in favor of the Triathlon all-polyethylene design, which may put patients at lower risk of aseptic loosening as compared with its metal-backed counterpart. However, smaller, non-significant differences in migration were found after adjustment for surgeon effect in the post hoc analysis. This unexpected surgeon effect warrants further investigation.

## Supplementary data

Tables 3, 4, and 5 and Figure 3 are available as supplementary data in the online version of this article, http://dx.doi.org/10.1080/17453674.2018.1464317

The study was designed and coordinated by STL. Data collection was performed by KH. Statistical analysis was done by KH and PM. KH, PM, RN, and STL interpreted the data and wrote the initial draft manuscript. All authors critically revised and approved the manuscript.

*Acta* thanks Stephan Maximilian Röhrl and other anonymous reviewers for help with peer review of this study.

## Supplementary Material

IORT_A_1464317_SUPP.pdf
