# Supplementary material for: Migration of all-polyethylene compared with metal-backed tibial components in cemented total knee arthroplasty: A randomized controlled trial
Source: Acta Orthop. 2018 May 1;89(4):412–7. doi: 10.1080/17453674.2018.1464317 (PMC6066763; doi:10.1080/17453674.2018.1464317)
Supplement: IORT_A_1464317_SUPP.pdf [file IORT_A_1464317_SM2737.pdf]

Supplementary data

Table 3. RSA migration analysis of mean absolute translation and rotation along and about each orthogonal axis (log-values are back-transformed in the original scale)

Translation/rotation	All-polyethylene mean (95% CI)	Metal-backed mean (95% CI)	p-value
Translation along transverse axis (mm)			
3 months	0.14 (0.09–0.20)	0.20 (0.14–0.25)	
1 year	0.14 (0.09–0.20)	0.22 (0.16–0.28)	
2 years	0.19 (0.14–0.25)	0.25 (0.19–0.31)	0.2
Translation along longitudinal axis (mm)			
3 months	0.12 (0.08–0.15)	0.11 (0.08–0.15)	
1 year	0.13 (0.09–0.16)	0.13 (0.10–0.17)	
2 years	0.10 (0.07–0.14)	0.15 (0.12–0.19)	0.08
Translation along sagittal axis (mm)			
3 months	0.19 (0.11–0.27)	0.19 (0.12–0.27)	
1 year	0.24 (0.16–0.32)	0.36 (0.27–0.45)	
2 years	0.25 (0.17–0.34)	0.43 (0.34–0.52)	0.006
Rotation about transverse axis (degrees)			
3 months	0.38 (0.27–0.49)	0.21 (0.12–0.30)	
1 year	0.48 (0.38–0.60)	0.38 (0.28–0.49)	
2 years	0.47 (0.36–0.59)	0.45 (0.34–0.57)	0.8
Rotation about longitudinal axis (degrees)			
3 months	0.18 (0.11–0.25)	0.19 (0.12–0.26)	
1 year	0.20 (0.13–0.27)	0.24 (0.17–0.31)	
2 years	0.20 (0.13–0.27)	0.29 (0.22–0.37)	0.09
Rotation about sagittal axis (degrees)			
3 months	0.26 (0.18–0.33)	0.23 (0.16–0.31)	
1 year	0.32 (0.25–0.41)	0.28 (0.21–0.36)	
2 years	0.34 (0.26–0.42)	0.33 (0.25–0.41)	0.8

Table 4. Post hoc sensitivity analysis of log-transformed maximum total point motion (logMTPM)

Effect	Mean difference in logMTPM between groups (95% CI)	p-value
Treatment effect (reference: all-polyethylene)		
3 months	–0.012 (–0.055 to 0.032)	
1 year	0.013 (–0.031 to 0.057)	
2 years	0.029 (–0.016 to 0.074)	0.2
Sex effect (reference: male)		
3 months	0.008 (–0.037 to 0.053)	
1 year	0.017 (–0.028 to 0.062)	
2 years	0.026 (–0.019 to 0.072)	0.3
Surgeon effect (reference: surgeon 1)		
3 months	0.080 (0.037 to 0.129)	
1 year	0.114 (0.070 to 0.157)	
2 years	0.129 (0.085 to 0.173)	< 0.001

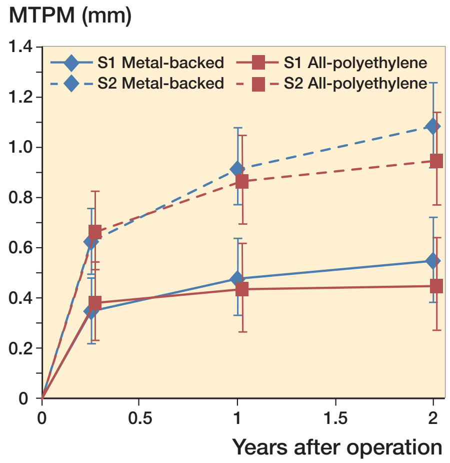

Figure 3. Post hoc sensitivity analysis results of maximum total point motion stratified by surgeon. The solid lines are the mean and 95% CI of the treatment groups of surgeon 1 (S1) and the dashed lines of surgeon 2 (S2).

Table 5. Functional outcomes. Values are mean and standard error in points, unless otherwise stated

Factor	All-poly-ethylene	Metal-backed	Difference in progression between groups mean (95% CI)	p-value
KSS Knee Score				
Preoperative	32.3 (2.9)	30.1 (2.8)		
3 months	85.6 (2.4)	78.3 (2.4)		
1 year	94.4 (1.8)	92.7 (1.7)		
2 years	91.9 (2.1)	93.4 (2.1)	3.7 (–4.6 to 12)	0.4
KSS Function Score				
Preoperative	58.8 (2.8)	57.5 (2.8)		
3 months	75.9 (2.6)	76.3 (2.6)		
1 year	90.1 (2.0)	87.3 (1.9)		
2 years	88.3 (2.8)	86.7 (2.7)	–0.3 (–8.3 to 7.7)	0.9
KOOS—Symptoms				
Preoperative	46.7 (2.5)	41.8 (2.5)		
3 months	51.6 (2.3)	51.7 (2.3)		
1 year	59.4 (2.6)	57.1 (2.5)		
2 years	62.1 (3.5)	61.8 (3.5)	4.6 (–5.9 to 15)	0.4
KOOS—Pain				
Preoperative	38.7 (3.3)	38.3 (3.4)		
3 months	69.8 (3.0)	60.5 (3.0)		
1 year	84.5 (3.0)	80.2 (2.9)		
2 years	79.2 (3.4)	83.2 (3.3)	4.5 (–5.5 to 14)	0.4
KOOS—ADL				
Preoperative	44.8 (3.3)	42.1 (3.3)		
3 months	69.9 (2.6)	64.2 (2.6)		
1 year	81.8 (2.7)	79.6 (2.7)		
2 years	79.4 (3.0)	80.5 (2.9)	3.8 (–5.4 to 13)	0.4
KOOS—Sports				
Preoperative	7.8 (1.9)	7.4 (2.0)		
3 months	19.5 (3.1)	21.7 (3.1)		
1 year	48.4 (4.3)	36.5 (4.2)		
2 years	41.5 (4.7)	41.3 (4.7)	0.2 (–13 to 14)	1.0
KOOS—QOL				
Preoperative	35.6 (1.5)	32.1 (1.6)		
3 months	46.1 (2.4)	44.5 (2.4)		
1 year	57.5 (2.8)	55.2 (2.7)		
2 years	57.5 (3.8)	57.8 (3.8)	3.7 (–7.3 to 15)	0.5
FJS				
3 months	38.4 (4.3)	30.9 (4.3)		
1 year	61.8 (4.8)	55.9 (4.7)		
2 years	56.9 (5.2)	57.5 (5.2)	8.1 (–5.4 to 21)	0.2

KSS: Knee Society Score; KOOS: Knee injury and Osteoarthritis Outcome Score; FJS: Forgotten Joint Score.
